# SOCS1 methylation level is associated with prognosis in patients with acute-on-chronic hepatitis B liver failure

**DOI:** 10.1186/s13148-023-01495-9

**Published:** 2023-05-06

**Authors:** Feng Li, Ying Zhang, Zhao-Hui Wang, Shuai Gao, Yu-Chen Fan, Kai Wang

**Affiliations:** 1 Department of Hepatology, Qilu Hospital of Shandong University, Wenhuaxi Road 107#, Jinan, 250012 Shandong China; 2 Hepatology Institute of Shandong University, Jinan, 250012 China

**Keywords:** Acute-on-chronic hepatitis B liver failure, DNA methylation, Glucocorticoid, MethyLight, Prognosis, SOCS1

## Abstract

**Background:**

Glucocorticoids could greatly improve the prognosis of patients with acute-on-chronic hepatitis B liver failure (ACHBLF). Suppressor of cytokine signaling (SOCS) 1 methylation has been shown to be associated with mortality in ACHBLF.

**Methods:**

Eighty patients with ACHBLF were divided into group glucocorticoid (GC) and group conservative medical (CM). Sixty patients with chronic hepatitis B (CHB), and Thirty healthy controls (HCs) served as control group. SOCS1 methylation levels in peripheral mononuclear cells (PBMCs) was detected by MethyLight.

**Results:**

SOCS1 methylation levels were significantly higher in patients with ACHBLF than those with CHB and HCs (*P* < 0.01, respectively). Nonsurvivors showed significantly higher SOCS1 methylation levels (*P* < 0.05) than survivors in both GC and CM groups in ACHBLF patients. Furthermore, the survival rates of the SOCS1 methylation-negative group were significantly higher than that of the methylation-positive group at 1 month (*P* = 0.014) and 3 months (*P* = 0.003) follow-up. Meanwhile, GC group and CM group had significantly lower mortality at 3 months, which may be related to application of glucocorticoid. In the SOCS1 methylation-positive group, the 1-month survival rate was significantly improved, which may be related to GC treatment (*P* = 0.020). However, no significant difference could be observed between the GC group and CM group in the methylation-negative group (*P* = 0.190).

**Conclusions:**

GC treatment could decrease the mortality of ACHBLF and SOCS1 methylation levels might serve as prognostic marker for favorable response to glucocorticoid treatment.

## Introduction

Hepatitis B virus (HBV) associated acute-on-chronic liver failure (ACLF), also defined as acute-on-chronic hepatitis B liver failure (ACHBLF), accounts for more than 70% of (ACLF) in China [1], which is an acute and rapid deterioration of liver function during the progression of diagnosed or undiagnosed chronic liver disease, manifesting as jaundice [serum bilirubin ≥ 5 mg/dL (85 μmol/L)] and coagulopathy (INR ≥ 1.5 or prothrombin activity < 40%), complicated within 4 weeks by ascites and/or encephalopathy [2, 3]. The disease progresses rapidly and has a very high mortality rate of 50–90% [4]. Liver transplantation remains the only definitive treatment for ACHBLF with limited application [5, 6]. Therefore, patients with ACHBLF urgently need treatment other than transplantation.

Immunologic imbalance plays a pivotal role in the pathophysiology of ACHBLF, including the activation of innate immune and the dysfunction of adaptive immune mediated by the T cell and cytokines, which contribute to the inflammation and necrosis of liver cells [7–10].

Corticosteroids can rapidly suppress excessive immune response and inflammatory response [11, 12] and have been proven to be an effective treatment for ACHBLF [13]. However, only some of the patients can benefit from corticosteroids treatment. Therefore, there is an urgent need for indicators that can evaluate the efficacy of corticosteroid therapy.

Suppressor of cytokine signaling (SOCS) 1 is a member of the SOCS protein family, including a center SH2 domain and a unique carboxyl SOCS box [14]. It has been identified as a crucial negative feedback regulator of various hematopoietic cytokines employing the Janus family of tyrosine kinases (JAK) and the signal transducers and activators of transcription (STAT) signaling [15]. SOCS1 protein limits the extent of Toll-like receptor signaling by inhibiting type I interferon (IFN) signaling [16, 17]. Accumulating evidences suggest that SOCS1 gene is frequently silenced by aberrant promoter methylation [18–20]. Previous studies suggested that cortisol might play a key role in suppressing cytokine signaling and inflammatory response through SOCS1 [21–23]. Therefore, there is a possibility that SOCS1 methylation level may reflect disease severity and predict prognosis of ACHBLF patients receiving glucocorticoid treatment.

## Materials and methods

### Participants

**Fig. 1:**
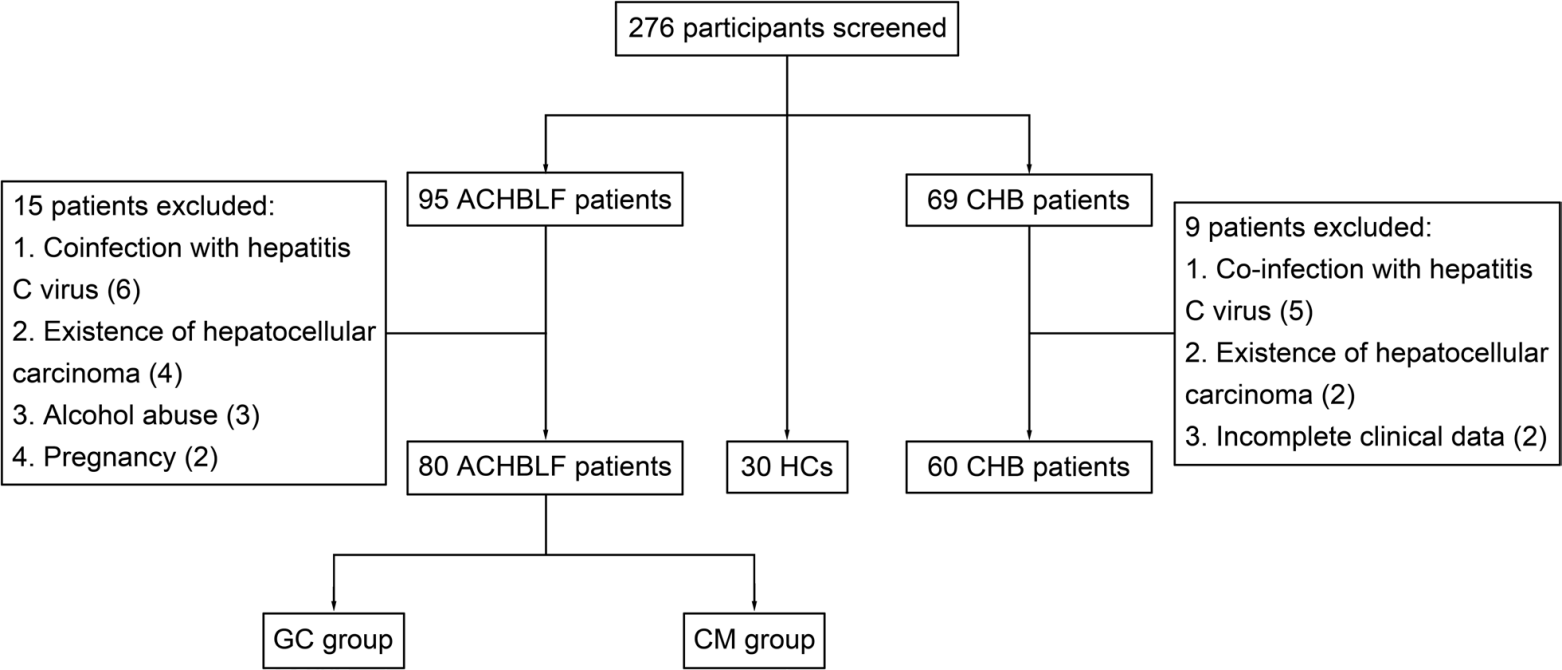
Flowchart for the enrollment of participants

Participants were recruited at Department of Hepatology, Qilu Hospital of Shandong University, July 2010 to March 2016. The research was permitted by the local Research and Ethics Committee at Qilu Hospital of Shandong University, in accordance with the guidelines of the 1975 Declaration of Helsinki. All participants gave their informed consent to participate in the study. The process of selecting participants has been shown in Fig. 1.

### Patients selection

The inclusion criteria were the following: (1) aged 18 years or older; (2) positive hepatitis B surface antigen (HBsAg) in serum for at least 6 months and (3) all enrolled patients met the criteria for 2009 update of American Association for the Study of Liver Diseases (AASLD) practice guidelines for management of chronic hepatitis B [24]. ACHBLF was diagnosed according to the consensus recommendations of Asian Pacific Association for the Study of the Liver (APASL) [2].

The exclusion criteria were the following: (1) severe infection (the amount of white blood cells > 10.0 × 10^9^/L and the percentage of neutrophile granulocyte > 70%); (2) combined with fungal infection, concomitant autoimmune or metabolic liver diseases, infection with hepatitis virus other than HBV, or human immunodeficiency virus, drug-induced hepatitis, alcoholic hepatitis; (3) pregnancy, liver tumors, complicated with alimentary tract hemorrhage, encephalopathy or massive ascites, and with other contraindication for steroid therapy. (4) used glucocorticoids during 6 months prior to this study.

### Treatment protocols

The patients were divided into conservative medical treatment (group CM) and glucocorticoid treatment (group GC), including 45 cases in GC group and 35 cases in CM group.

Conservative treatments included antiviral therapy, absolute bed rest, nutritional support, hepatoprotective drugs and prevention and treatment of complications, etc. All conservative medical management were performed based on the criteria of APASL consensus recommendations [2, 25].

The GC group received 0.75 mg/(kg day) (average: 60 mg/day) of prednisolone for the first 3 days, then 0.5 mg/(kg day) (average: 40 mg/day) of prednisolone for the second 3 days and followed by 0.25 mg/(kg day) (average: 20 mg/day) of prednisolone until the end of the third 3-day period. Depending on the improvement in liver function, the dose of prednisolone was gradually reduced by 5 mg at least every 4 days until the drug was completely discontinued on the 28th day [13].

### Study outcomes

The follow-up date was started at the onset of glucocorticoid treatment. We used 3-month mortality as the primary prognosis and 1-month mortality was also used for short-term prognosis.

### DNA extraction and sodium bisulphite modification

According to the instructions, peripheral blood mononuclear cells (PBMCs) was isolated by density gradient centrifugation with Ficoll-Paque (Pharmacia Diagnostics, Uppsala, Sweden) and stored at − 20 °C. Genomic DNA was extracted from PBMC by QIAamp DNA Blood Mini Kit (QIAGEN, Valencia, CA, USA) following the standard protocol. DNA samples were eluted in 200 μL sterile water and stored at − 20 °C until further processing.

The extracted DNA was treated with sodium bisulfite using the EZ DNA Methylation-Gold Kit (Zymo Research, Orange, CA, USA) following the manufacturer’s protocol. The modified DNA was either used immediately as a template for MethyLight or stored at − 20 °C.

### Taqman probe-based quantitative methylation specific polymerase chain reaction (MethyLight)

MethyLight was used to detect the methylation level of SOCS1 promoter in all participants. The SOCS1 [26] and Alu-C4 [27] gene specific primers and probes were designed as previous published studies (Table 1). We used a total volume of 10 μl containing 5.5 μL nuclease-free water, 2 μL buffer (LightCycler TaqMan Master; Roche Diagnostics, Mannheim, Germany) consisted of FastStart Taq DNA polymerase, reaction buffer, MgCl_2_ and deoxynucleotide triphosphate mixture, 0.5 μL of forward and reverse primers (300 nmol/L), 0.25 μL Taqman probe (150 nmol/L) and 1.25 μL bisulphite- converted DNA. MethyLight was performed on Lightcycler 2.0 (Roche, Basel, Switzerland) following the standard protocol provided by manufacturer [28, 29]: 95 °C for 10 min, then 50 cycles of 95 °C for 15 s and 60 °C for 1 min.

**Table 1:** Primer and Taqman probe sequences used to amplify bisulphite converted DNA in MethyLight analysis

Gene	Primer and Taqman probe sequences (5′–3′)
*SOCS1*	Forward: GCGTCGAGTTCGTGGGTATTT Reverse: CCGAAACCATCTTCACGCTAA Probe: 6FAM-ACAATTCCGCTAACGACTATCGCGCA-TAMRA
*Alu-C4*	Forward: GGTTAGGTATAGTGGTTTATATTTGTAATTTTAGTA Reverse: ATTAACTAAACTAATCTTAAACTCCTAACCTCA Probe: 6FAM-CCTACCTTAACCTCCC-TAMRA

Universal methylated and bisulphite- converted human control DNA (QIAGEN, Hilden, Germany) served as a methylated reference and Alu-C4 was used as a control reaction to normalise for input DNA. The MethyLight data were expressed as percent of methylated reference (PMR) values [28, 29]. PMR is an acronym for percent of methylated reference. Ct value: C represents Cycle and t represents threshold. The meaning of Ct value is the number of cycles that fluorescence signals in each reaction tube go through when they reach the set domain value. PMR values ≥ 4% were defined as methylation-positive and PMR values < 4% indicated methylation-negative [26, 30].

$$\begin{aligned} {\text{PMR}} & { = }100\% \times 2 exp - \left[ {\Delta Ct \left( {target\;gene\;in\;sample - control\;gene\;in\;sample} \right)} \right. \\ & \quad \left. { - \Delta Ct \left( {100\% methylated\;target\;in\;reference\;sample - control\;gene\; in\;reference\;sample} \right)} \right] \\ \end{aligned}$$

Ct is the threshold cycle.

### Clinical parameters

The serum biochemical markers (COBAS integra 800, Roche Diagnostics, Penzberg, Germany) included alanine aminotransferase (ALT), aspartate aminotransferase (AST), total bilirubin (TBIL), albumin (ALB) and creatinine (Cr). Hemostasis markers (ACL TOP 700; Instrument Laboratory, Lexington, MA, USA) included prothrombin time-international normalised ratio (PT-INR) and prothrombin time activity (PTA). Hepatitis B e antigen (HBeAg) was assayed using an automatic analyzer (Cobas 6000 analyzer series; Roche Diagnostics, Basel, Switzerland). Serum HBV-DNA was quantified using a polymerase chain reaction (PCR) System (ABI 7300; Applied Biosystems, Foster City, CA, USA) with a detection sensitivity of 500 IU/mL. These markers were measured using operating procedure in Department of Medicine Laboratory, Qilu Hospital, Shandong University. Model for end-stage liver disease (MELD) score was calculated according to the original formula [31].

$$\begin{aligned} {\text{MELD}}\;{\text{score}} & = \left[ {9.57 \times log_{e} creatinine + 3.78 \times log_{e} bilirubin + 11.20 \times log_{e} INR} \right. \\ & \quad \left. { + 6.43\left( {constant\;for\;liver\;disease\;etiolog} \right)} \right] \\ \end{aligned}$$

creatinine mg⁄dL, bilirubin mg⁄dL

### Statistical analysis

Quantitative variables were expressed as median (centile 25; centile 75). Categorical variables were expressed as number (percentage). The data were analyzed using SPSS 16.0 statistical software (SPSS Inc., Chicago, IL, USA). Student’s t test and Mann–Whitney U test were used to compare the quantitative variables. Chi-square test was used to compare the categorical variables. The relationship between SOCS1 methylation level and clinicopathological data was evaluated using the spearman rank correlation test. The area under the receiver operating characteristic curve (AUROC) was used to assess the diagnostic value of SOCS1 methylation in predicting mortality of ACHBLF. From the receiver operating characteristic (ROC) curve coordinates, cut-off points with best sensitivity and specificity were selected. Diagnostic accuracy was assessed by sensitivity, specificity, positive predictive value (PPV) and negative predictive value (NPV). Our target variable is whether the patient died or not. The survival of patients at 1 month and 3 months were counted. If the patient had not died by the end of the trial, it could be considered censored data. Survival curve was drawn using the Kaplan–Meier method and the statistical significance was determined using log-rank test. All statistical analyses were two sided. The difference was statistically significant (*P* < 0.05).

## Results

### General characteristics

The process of study selection and exclusion was shown in Fig. 1. A total of 164 patients and 30 healthy controls were evaluated. After exclusion of 24 patients who did not meet the inclusion criteria, 170 cases were enrolled. Table 2 shows the basic characteristics for both groups. There was no significant difference between treatment and control groups at the baseline characteristics.

**Table 2:** The basic characteristics of all the enrolled participants

Variable	ACHBLF group (n = 80)		CHB group (n = 60)	HC group (n = 30)
	GC group (n = 45)	CM group (n = 35)		
Male (%)	31 (68.9)	25 (71.4)	43 (61.4)	19 (63.3)
Age (years)	44.0 (32.0–51.5)	45.0 (37.0–57.0)	42.0 (34.0–48.5)	36.5 (28.3–42.8)
HBeAg + (%)	20 (44.4)	18 (51.4)	28 (46.7)	NA
Log_10_ [HBV DNA]	4.3 (3.3–5.9)	4.3 (3.9–6.7)	4.5 (3.2–5.5)	NA
ALT (U/L)	238.0 (136.5–513.0)	150.0 (101.0–373.0)	90.0 (56.5–148.5)	18.0 (12.8–23.0)
AST (U/L)	164.0 (94.0–310.5)	130.0 (85.0–179.0)	65.5 (38.5–91.5)	18.0 (15.8–23.0)
TBIL (mg/dL)	12.5 (10.8–19.4)	13.3 (10.4–22.8)	0.9 (0.7–1.1)	0.7 (0.5–0.8)
ALB (g/L)	30.8 (28.6–36.0)	32.7 (28.9–35.7)	42.8 (40.4–45.9)	49.5 (47.2–51.3)
Cr (mg./dL)	0.7 (0.6–0.9)	0.8 (0.6–0.9)	0.7 (0.6–0.8)	0.7 (0.6–0.8)
INR	1.7 (1.6–1.9)	1.8 (1.6–2.1)	1.0 (1.0–1.1)	1.0 (1.0–1.1)
PTA (%)	35.0 (30.0–37.5)	34.0 (30.0–38.0)	83.5 (73.5–97.0)	96.0 (88.8–104.0)
MELD score	19.8 (18.2–22.8)	20.6 (18.7–25.1)	NA	NA
*SOCS1* methylation level	23.8 (7.9–61.6)	34.2 (2.4–71.7)	1.8 (1.0–3.0)	0.8 (0.6–1.3)

*ACHBLF* acute-on-chronic hepatitis B liver failure, *CHB* chronic hepatitis B, *HCs* healthy controls, *GC* glucocorticoid, *CM* conservative medical, *HBeAg* hepatitis B e antigen, *ALT* alanine aminotransferase, *AST* aspartate aminotransferase, *TBIL* total bilirubin, *ALB* albumin, *Cr* creatinine, *INR* international normalized ratio, *PTA* prothrombin time activity, *MELD* model for end-stage liver disease, *NA* not available

Quantitative variables were expressed as the median (centile 25; centile 75). Categorical variables were expressed as number (%)

### Hypermethylation of SOCS1 promoter in patients with ACHBLF

**Fig. 2:**
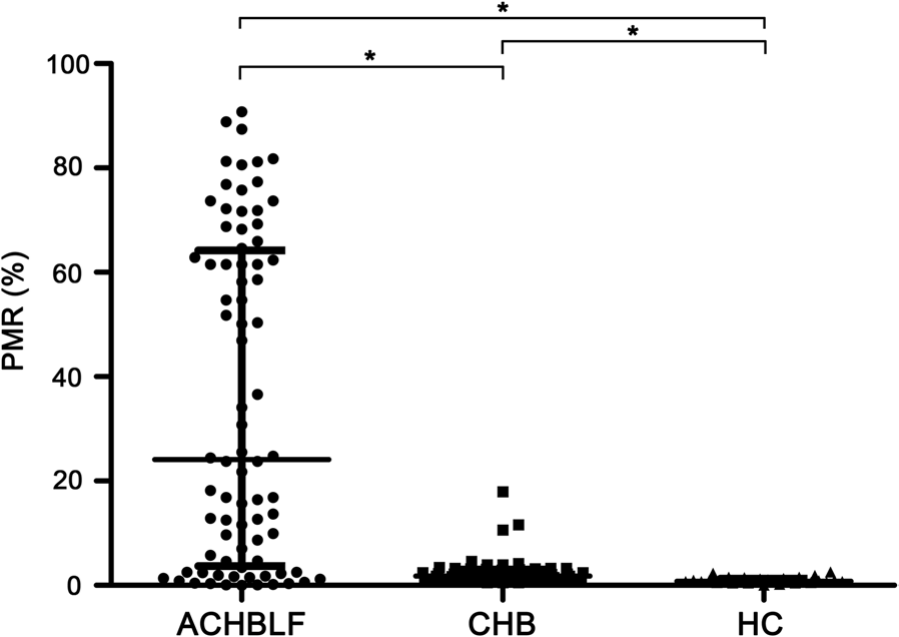
The SOCS1 methylation levels in different participants groups. SOCS1 methylation level was significantly higher in patients with ACHBLF than in those with CHB (*P* < 0.01) and HCs group (*P* < 0.01).

The methylation status of SOCS1 promoter expressed as PMR in ACHBLF, CHB and HCs groups respectively were presented in Fig. 2. As shown in the figure, SOCS1 methylation levels in patients with ACHBLF (median 24.15, interquartile range 3.72–64.18) were significantly higher than that in CHB (median 1.77, interquartile range 0.98–3.01; *P* < 0.01) and HCs (median 0.82, interquartile range 0.59–1.25; *P* < 0.01). In addition, the SOCS1 methylation levels of CHB patients were significantly higher than HCs (*P* < 0.01). No significant difference could be found between SOCS1 methylation levels in the GC and CM groups.

### Associations between SOCS1 promoter methylation level and clinicopathological features in ACHBLF patients

**Fig. 3:**
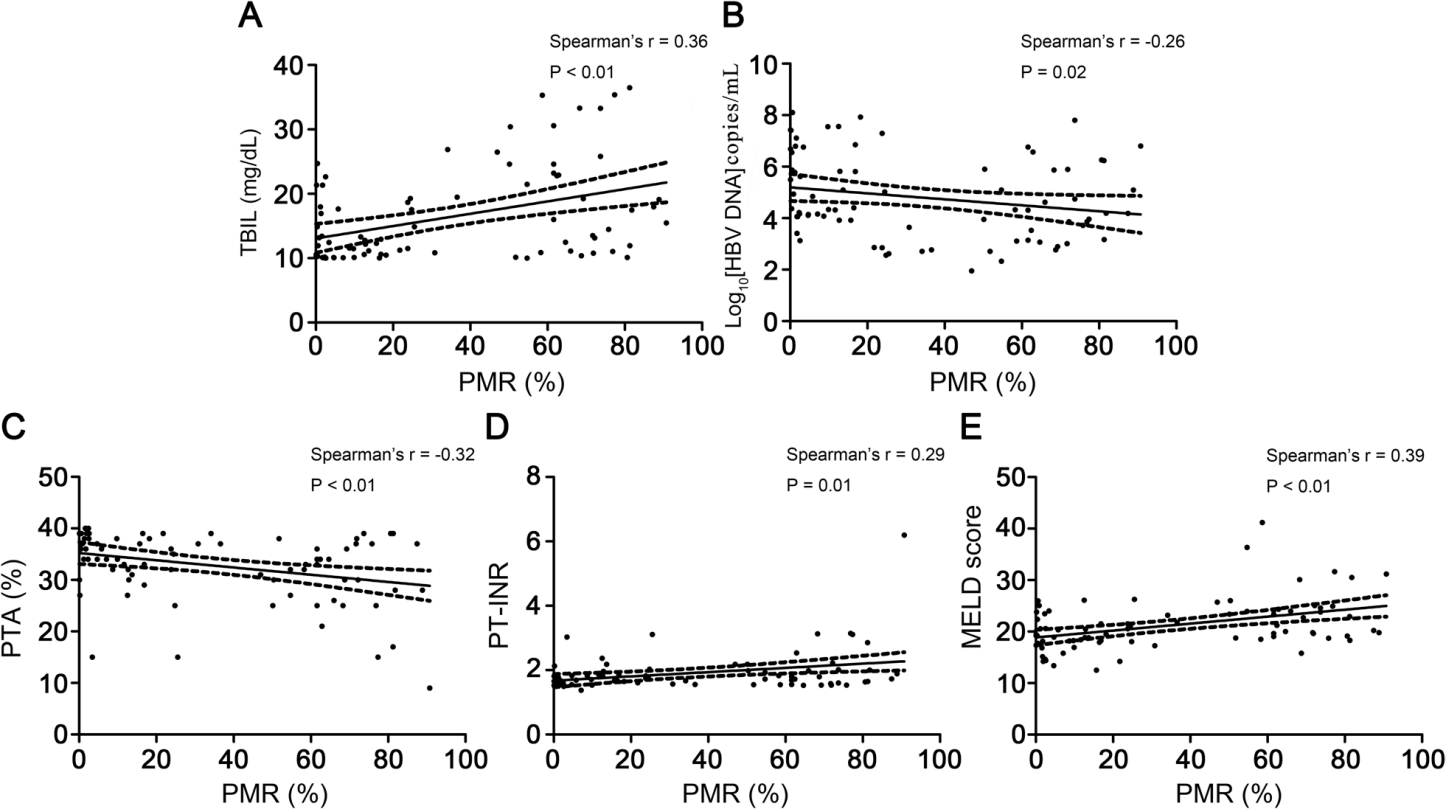
Associations between SOCS1 promoter methylation level and clinicopathological features in ACHBLF patients **A** Correlation between PMR value of SOCS1 promoter and TBIL level (Spearman’s r = 0.36, *P* < 0.01). **B** Correlation between PMR value of SOCS1 promoter and HBV DNA load (Spearman’s r = − 0.26, *P* = 0.02). **C** Correlation between PMR value of SOCS1 promoter and PTA level (Spearman’s r = − 0.32, *P* < 0.01). **D** Correlation between PMR value of SOCS1 promoter and PT-INR level (Spearman’s r = 0.29, *P* = 0.01). **E** Correlation between PMR value of SOCS1 promoter and MELD score (Spearman’s r = 0.39, *P* < 0.01). TBIL, total bilirubin; INR, international normalized ratio; PTA, prothrombin time activity; MELD, model for end-stage liver disease

We found that SOCS1 methylation levels were significantly correlated with TBIL (Spearman’s r = 0.36, *P* < 0.01), PTA (Spearman’s r = − 0.32, *P* < 0.01), PT-INR (Spearman’s r = 0.29, *P* = 0.01), log10[HBV DNA] (Spearman’s r = − 0.26, *P* = 0.02) and MELD score (Spearman’s r = 0.39, *P* < 0.01). However, there was no association between SOCS1 promoter methylation levels and Age (Spearman’s r = − 0.09, *P* = 0.41), ALT (Spearman’s r = − 0.11,* P* = 0.32), AST (Spearman’s r = − 0.04, *P* = 0.74), ALB (Spearman’s r = − 0.13, *P* = 0.24), Cr (Spearman’s r = 0.15, *P* = 0.17) (Fig. 3A–E).

### One and three-month SOCS1 methylation levels analysis in ACHBLF survivors

**Fig. 4:**
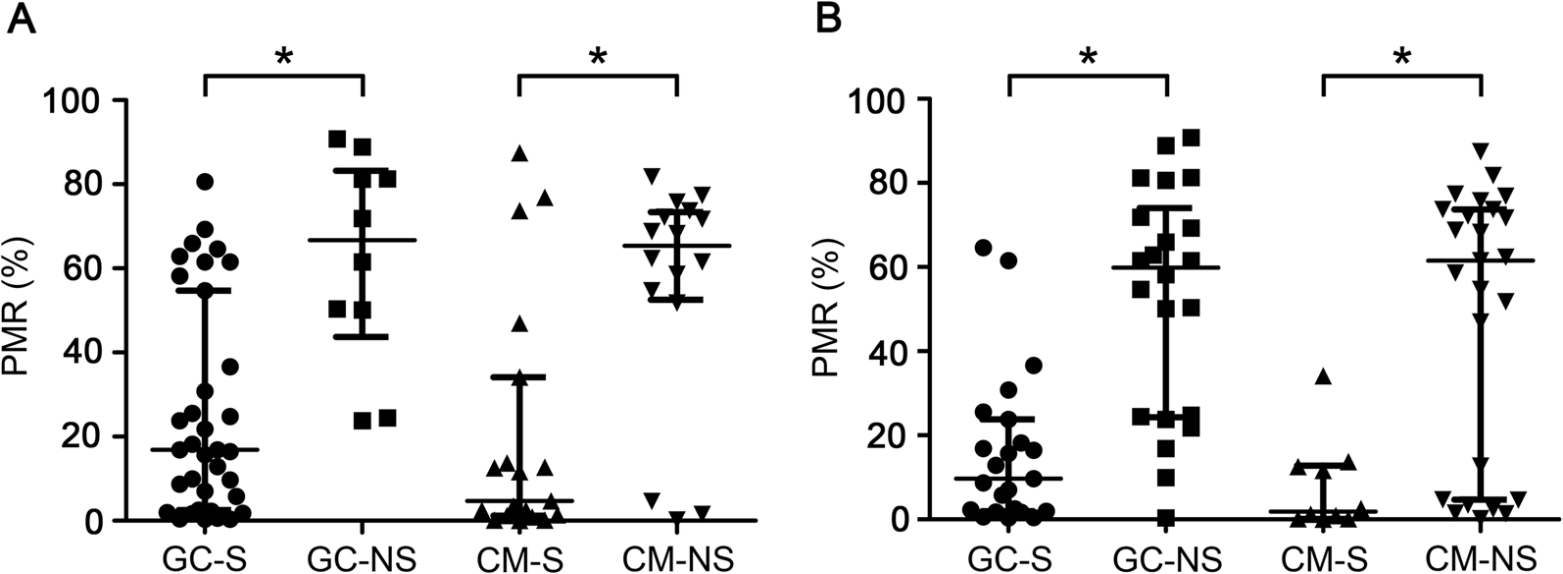
One and three-month SOCS1 methylation levels analysis in ACHBLF survivors. **A** The PMR value of SOCS1 promoter in survivors and non-survivors at the end of 1-month. **B** The PMR value of SOCS1 promoter in survivors and non-survivors at the end of 3-month. GC, glucocorticoid; CM, conservative medical; S, survivors; NS, non-survivors

At the 1-month, 26 patients had died and the mortality rate was 32.5%.SOCS1 methylation levels of nonsurvivors at baseline were significantly higher than those of survivors in both the GC group and the CM group (GC group: [median 16.84, interquartile range 2.52–54.71] for survivor vs. [median 66.71, interquartile range 43.71–83.21] for nonsurvivors, *P* < 0.01; CM group: [median 4.64, interquartile range 1.17–13.68] for survivor vs. [median 65.36, interquartile range 52.50–73.33] for nonsurvivors, *P* < 0.01) (Fig. 4A). At the 3-month, 47 patients had died and the mortality rate was 58.75%. SOCS1 methylation levels of nonsurvivors at baseline were significantly higher than those of survivors in both the GC group and the CM group (GC group: [median 9.74, interquartile range 1.92–21.76] for survivor vs. [median 59.90, interquartile range 24.74–74.06] for nonsurvivors, *P* < 0.01; CM group: [median 1.96, interquartile range 0.11–12.79] for survivor vs. [median 61.56, interquartile range 4.67–73.71] for nonsurvivors, *P* < 0.01) (Fig. 4B).

### Hypermethylation of SOCS1 promoter as a predictor for poor prognosis of ACHBLF patients

**Fig. 5:**
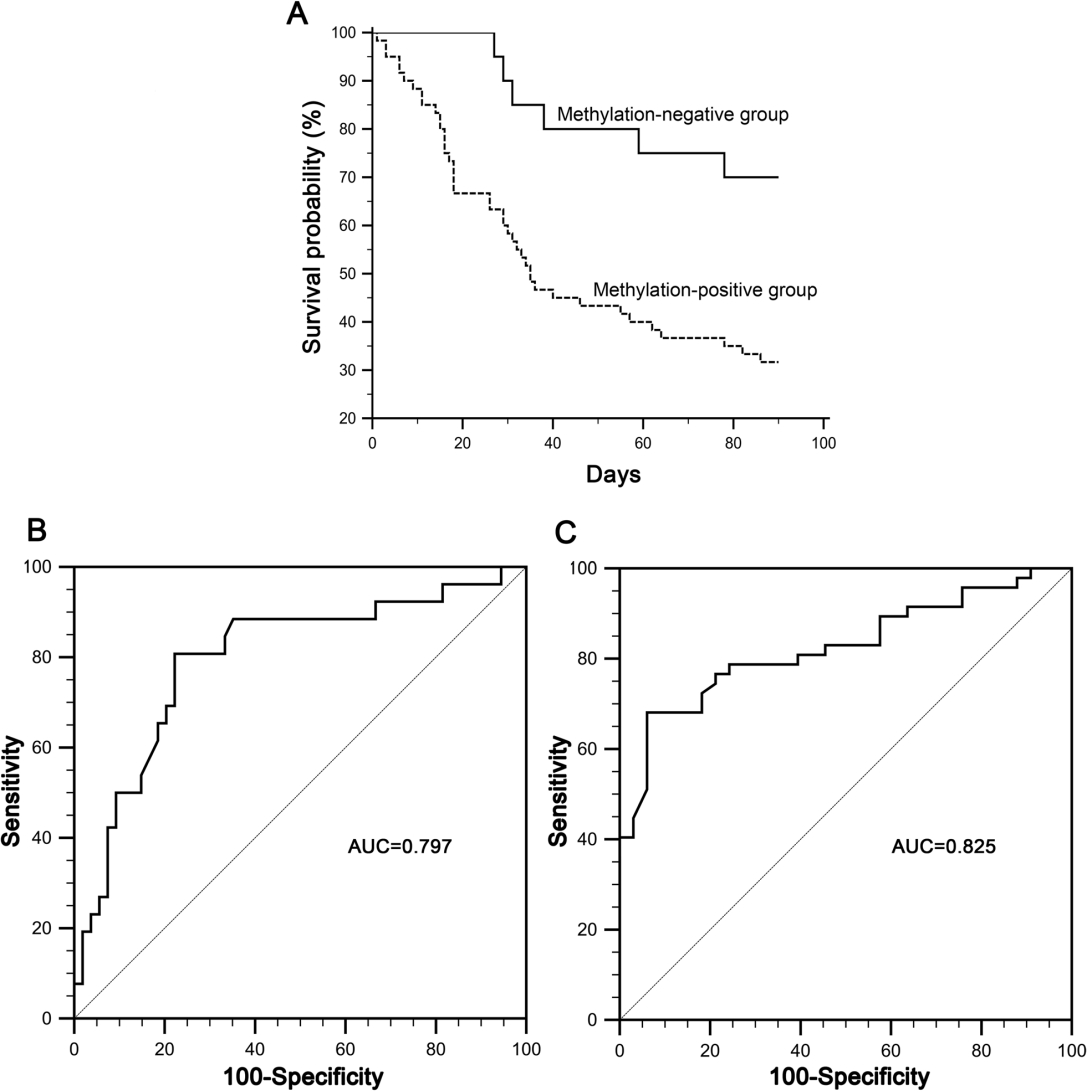
Kaplan–Meier curves and Receiver operating characteristic (ROC) curves for SOCS1 methylation level in ACHBLF patients. **A** Kaplan–Meier curves for the SOCS1 methylation-positive and negative group. **B** ROC curves of SOCS1 methylation level in predicting 1-month mortality of ACHBLF patients. (c) ROC curves of SOCS1 methylation level in predicting 3-month mortality of ACHBLF patients

According to PMR values, ACHBLF patients were divided into methylation-positive group and methylation-negative group. We found that the survival rates of the SOCS1 methylation-negative group was significantly higher than that of the methylation-positive group at the end of 3 months (*P* = 0.003) follow-up (Fig. 5A). Receiver operating characteristic (ROC) curve was used to estimate the diagnostic value of SOCS1 methylation levels in predicting 1-month mortality for ACHBLF patients. As shown in Fig. 5B, the AUROC was 0.797 (S.E. 0.057, 95% CI 0.693–0.879) for SOCS1 methylation levels. A cut-off value of 46.98% with sensitivity of 80.77%, specificity of 77.78%, positive predictive value (PPV) of 63.6%, and negative predictive value (NPV) of 89.4% was selected to discriminate survivals and nonsurvivors at the end of 1-month follow-up. As shown in Fig. 5C, the AUROC was 0.825 (S.E. 0.046, 95% CI 0.724–0.901) for SOCS1 methylation levels. A cut-off value of 36.60% with sensitivity of 68.09%, specificity of 93.94%, positive predictive value (PPV) of 94.1%, and negative predictive value (NPV) of 67.4% was selected to discriminate survivals and nonsurvivors at the 3-month follow-up.

### Improved survival ratios correlate with GC treatment in ACHBLF patients

**Fig. 6:**
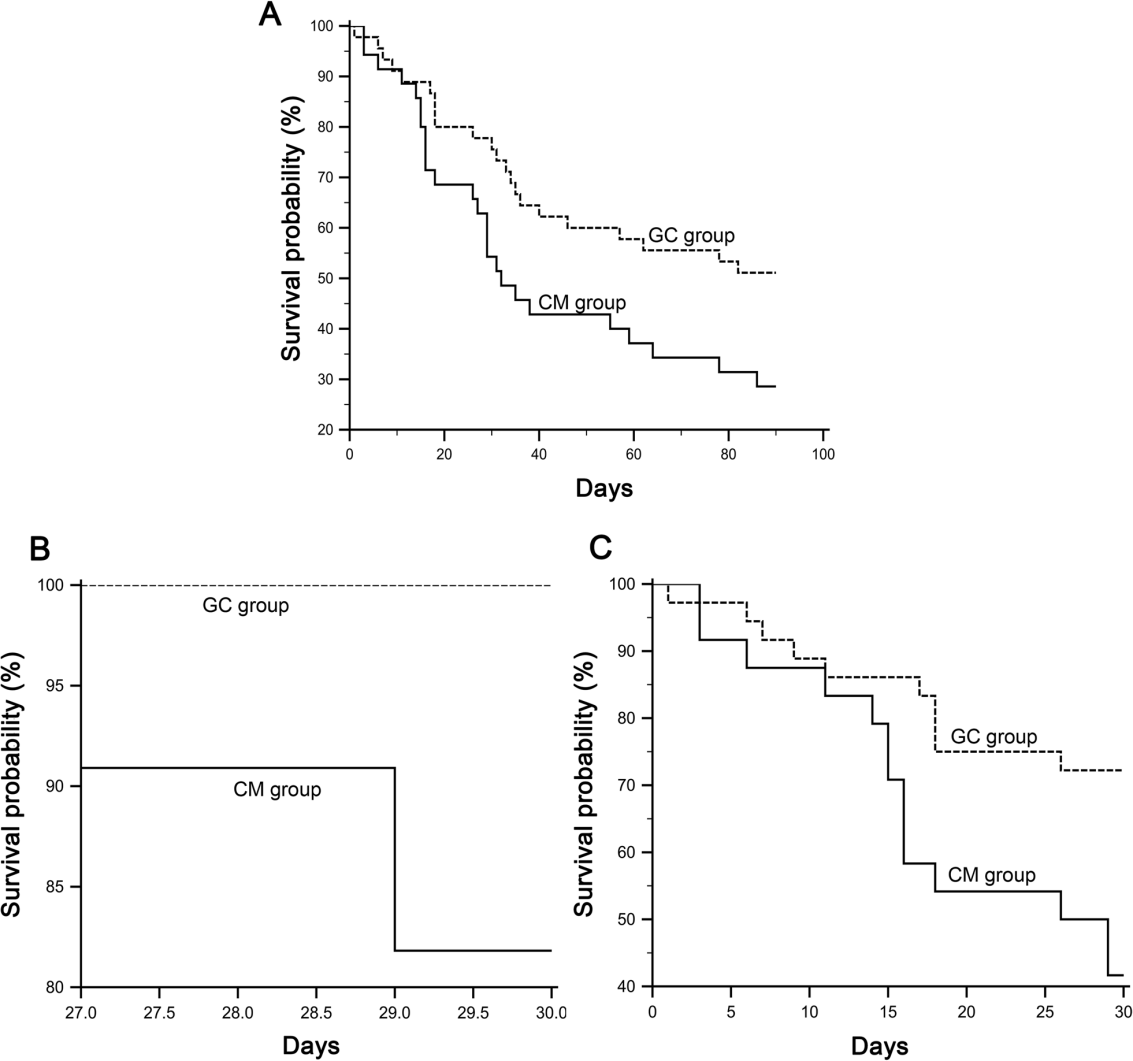
Kaplan–Meier curves for glucocorticoid (GC) and conservative medical management (CM) therapy. **A** Kaplan–Meier curves for GC and CM therapy. **B** Kaplan–Meier curves for GC and CM therapy in methylation-positive group. **C** Kaplan–Meier curves for GC and CM therapy in methylation-negative group

We monitored further the effect of GC treatment on the survival rates of enrolled patients in this study. At the 3-month (log-rank test with *P* = 0.033), GC group showed significantly higher survival rates than those in CM group (Fig. 6A). These data suggest that GC therapy was associated with increased survival in ACHBLF patients.

### Lower SOCS1 methylation level as a predictor for favorable response to glucocorticoid treatment in ACHBLF patients

We investigate the association of SOCS1 methylation level with the response to glucocorticoid treatment in patients with ACHBLF. GC group had significantly higher 1-month survival rates than CM group in the SOCS1 methylation-positive group (*P* = 0.020). No significant difference could be observed between the 1-month survival rates in GC and CM groups in the SOCS1 methylation-negative group (*P* = 0.190) (Fig. 6B, C).

## Discussion

In this study, we found that SOCS1 methylation levels were significantly higher in patients with ACHBLF than those with CHB and HCs. In both GC and CM groups, nonsurvivors had significantly higher SOCS1 methylation levels (*P* < 0.05) than survivors in ACHBLF patients. Meanwhile, after 1-month (*P* = 0.014) and 3-month (*P* = 0.003) follow-up, SOCS1 methylation-positive patients showed significantly poorer survival compared with methylation-negative group. Furthermore, GC treatment may be associated with decreased mortality at 1 and 3 months in ACHBLF patients. In the SOCS1 methylation-positive group, the 1-month survival rate was significantly improved, which may be related to GC treatment. However, there was no significant difference between the GC group and the CM group in the methylation-negative group (*P* = 0.190).

DNA methylation refers to the addition of a methyl group to DNA, which is one of the most important epigenetic mechanisms. Previous studies have shown that enhanced demethylation of IFN-γ gene promoter in PBMC may be related to the occurrence of ACHBLF [32]. Hypermethylation of SOCS1, associated with the response to corticosteroid treatment, had been detected in patients with ACHBLF [13]. However, most previous studies used methylation-specific polymerase chain reaction (MSP), which was just a qualitative method to identify whether methylation has occurred [26, 33]. In this study, we use MethyLight to quantitatively detect the methylation level of target genes. The Methylight has quantitative and high-throughput properties and relatively simple analysis procedures, which are extremely beneficial in clinical molecular diagnostics. Compared with traditional MSP widely used in methylation detection, MethyLight has higher sensitivity [34, 35].

ACLF is an acute and severe deterioration of liver function in a patient with chronic liver disease [2, 4]. In China, ACLF is mainly caused by hepatitis B virus (HBV) infection [36]. Although the mechanisms of hepatic failure are not fully elucidated, immunologically mediated events play an important role in the pathogenesis of ACHBLF. Accumulating evidences suggest that several pro-inflammatory cytokines may play pivotal roles in ACHBLF and the hyperactivated immune responses mediated by pro-inflammatory cytokines bring about hepatic inflammation and necrosis [10, 37]. As an immunomodulator, corticosteroids can quickly suppress excessive immune response and inflammatory response. It has been proven to be effective in treating non-viral hepatitis-related liver failure and severe deterioration of chronic hepatitis B that may be life-threatening [11, 38]. Previous studies shown that in the early stages of severe hepatitis, the use of corticosteroids is essential to prevent liver cell necrosis [39]. Therefore, it is a reasonable treatment decision to treat patients with ACHBLF with corticosteroids to suppress excessive immune response and prevent infection of hepatocytes. However, this therapy has not yet shown satisfactory clinical effects and has not yet been determined [12, 40]. An accurate and simple prognostic factor is urgently needed to guide and optimize the treatment of ACHBLF.

SOCS1 is considered to be a key negative feedback regulator of cytokine stimulation and is extremely important for limiting the inflammatory response. We have previously demonstrated that SOCS1 plays an important role in the pathogenesis of ACHBLF. In this study, we found that compared with CHB and HCs, SOCS1 methylation levels in PBMCs of patients with ACHBLF were significantly increased. In addition, SOCS1 methylation levels in ACHBLF patients are significantly increased, and are positively correlated with serum TBIL, PTA, and MELD scores, which are often used as markers of liver injury in ACHBLF patients. Furthermore, we also reported that compared with survivals, nonsurvivals had higher levels of SOCS1 methylation, and SOCS1 was associated with the mortality of ACHBLF patients. These results strongly suggest that SOCS1 may be involved in the pathogenesis of ACHBLF, and further suggest that the level of SOCS1 methylation may determine the prognosis of patients with ACHBLF.

Corticosteroids was used to improve the outcome of liver failure due to hepatotoxic drugs or autoimmune hepatitis. However, the efficacy of glucocorticoid therapy in patients with ACHBLF remains controversial. It has been proven that methylprednisolone treatment can improve the 28-day survival rate of patients with ACHBLF [41]. Our research shows that the survival rate of ACHBLF patients in the GC group was significantly higher than that of the CM group at 1 month and 3 months follow-up. These results indicated that. GC therapy was associated with increased survival in ACHBLF patients. Importantly, we evaluate the relationship between SOCS1 methylation levels and the efficacy of GC treatment for patients with ACHBLF. GC patients had significantly higher survival rates compared with CM patients in the SOCS1 methylation-positive group while not in the SOCS1 methylation-negative group. This may represent a favorable response to corticosteroid treatment in ACHBLF patients with high SOCS1 promoter methylation level.

In summary, we demonstrated GC therapy was associated with increased survival in ACHBLF patients. Meanwhile, we found that there was a potential role for SOCS1 methylation level in the prediction of prognosis in these patients. Current treatments for liver failure are limited. Liver transplantation is the only definitive therapeutic option for patients with ACHBLF. However, few patients can benefit from this approach because of the great imbalance between donation and potential recipients and the high cost of the procedure. And at present, there is no suitable method to predict the therapeutic effect of corticosteroids. Our study demonstrates the effectiveness of corticosteroids therapy and provide a better therapeutic option for the treatment of ACHBLF. More importantly, using SOCS1 methylation levels to evaluate the efficacy of glucocorticoids in the treatment of ACHBLF can predict at the molecular level which part of ACHBLF patients will benefit from glucocorticoids. On the one hand, it can guide doctors' medication and select appropriate liver failure patients for treatment, so as to help patients win precious treatment time. On the other hand, it can save medical resources.

However, there are still several limitations in this study. Firstly, we did not analyze the intrahepatic SOCS1 methylation status of the studied patients because it was virtually impossible to carry liver biopsy in patients with ACHBLF who had coagulopathy and high bleeding risk. Secondly, the sample size was relatively small and all patients were selected from single center, which might lead to selection bias. In the future, follow-up studies include a multi-center, larger and prospective cohort were needed to confirm our current findings. Meanwhile, the precise molecular mechanism about how SOCS1 was involved in the progress of ACHBLF remained unclear and might also be studied in our further study.

## Data Availability

The datasets used and/or analyzed during the current study are available from the corresponding author on reasonable request.
